# Effect of flow change on brain injury during an experimental model of differential hypoxaemia in cardiogenic shock supported by extracorporeal membrane oxygenation

**DOI:** 10.1038/s41598-023-30226-6

**Published:** 2023-03-10

**Authors:** Sacha Rozencwajg, Silver Heinsar, Karin Wildi, Jae‐Seung Jung, Sebastiano Maria Colombo, Chiara Palmieri, Kei Sato, Carmen Ainola, Xiaomeng Wang, Gabriella Abbate, Noriko Sato, Wayne B. Dyer, Samantha Livingstone, Leticia Helms, Nicole Bartnikowski, Mahe Bouquet, Margaret R. Passmore, Kieran Hyslop, Bruno Vidal, Janice D. Reid, Daniel McGuire, Emily S. Wilson, Indrek Rätsep, Roberto Lorusso, Matthieu Schmidt, Jacky Y. Suen, Gianluigi Li Bassi, John F. Fraser

**Affiliations:** 1 Service de Réanimation Médicale, Groupe Hospitalier Pitié–Salpêtrière, Institut de Cardiologie, Assistance Publique–Hôpitaux de Paris, Hôpital de la Pitié–Salpêtrière, 47, bd de l’Hôpital, 75651 Paris Cedex 13, France; 2 UPMC Université Paris 06, INSERM, UMRS-1166, ICAN Institute of Cardiometabolism and Nutrition, Sorbonne Universités, Paris, France; 3 Critical Care Research Group, The Prince Charles Hospital, Level 3, Clinical Sciences Building, Chermside, Brisbane, QLD 4032 Australia; 4 Present Address: Faculty of Medicine, University of Queensland, Brisbane, Australia; 5 Department of Intensive Care, North Estonia Medical Centre, Tallinn, Estonia; 6 Department of Thoracic and Cardiovascular Surgery, College of Medicine, Korea University, Seoul, Republic of Korea; 7 Department of Pathophysiology and Transplantation, University of Milan, Milan, Italy; 8 Present Address: Department of Anaesthesia and Intensive Care Medicine, Fondazione IRCCS Ca’ Granda Ospedale Maggiore Policlinico, Milan, Lombardia Italy; 9 Australian Red Cross Lifeblood, Sydney, Australia; 10 Science and Engineering Faculty, Queensland University of Technology, Brisbane, QLD Australia; 11 Cardio-Thoracic Surgery Department, Maastricht University Medical Centre, Maastricht, The Netherlands; 12 Queensland University of Technology, Brisbane, Australia; 13 Present Address: Intensive Care Unit, St Andrew’s War Memorial Hospital, Brisbane, Australia; 14 Intensive Care Unit, The Wesley Hospital, Brisbane, Australia; 15 Columbia University, College of Physicians and Surgeons, New York, USA; 16 Wesley Medical Research, The Wesley, Queensland Auchenflower, Australia

**Keywords:** Circulation, Physiology, Cardiology

## Abstract

Differential hypoxaemia (DH) is common in patients supported by femoral veno-arterial extracorporeal membrane oxygenation (V-A ECMO) and can cause cerebral hypoxaemia. To date, no models have studied the direct impact of flow on cerebral damage. We investigated the impact of V-A ECMO flow on brain injury in an ovine model of DH. After inducing severe cardiorespiratory failure and providing ECMO support, we randomised six sheep into two groups: low flow (LF) in which ECMO was set at 2.5 L min^−1^ ensuring that the brain was entirely perfused by the native heart and lungs, and high flow (HF) in which ECMO was set at 4.5 L min^−1^ ensuring that the brain was at least partially perfused by ECMO. We used invasive (oxygenation tension—PbTO_2_, and cerebral microdialysis) and non-invasive (near infrared spectroscopy—NIRS) neuromonitoring, and euthanised animals after five hours for histological analysis. Cerebral oxygenation was significantly improved in the HF group as shown by higher PbTO_2_ levels (+ 215% vs − 58%, *p* = 0.043) and NIRS (67 ± 5% vs 49 ± 4%, *p* = 0.003). The HF group showed significantly less severe brain injury than the LF group in terms of neuronal shrinkage, congestion and perivascular oedema (*p* < 0.0001*)*. Cerebral microdialysis values in the LF group all reached the pathological thresholds, even though no statistical difference was found between the two groups. Differential hypoxaemia can lead to cerebral damage after only a few hours and mandates a thorough neuromonitoring of patients. An increase in ECMO flow was an effective strategy to reduce such damages.

## Introduction

Veno-arterial extracorporeal membrane oxygenation (V-A ECMO) is an increasingly used rescue therapy for patients with refractory cardiogenic shock (CS)^1,2^. It provides mechanical circulatory support for both heart and lungs by generating blood flow, oxygenating, and removing carbon dioxide. Although a life-saving therapy, V-A ECMO exposes patients to several complications, in particular bleeding, infections, and cerebrovascular accidents^3^. Peripheral femoral cannulation (the most used configuration) also brings a physiological paradigm with a retrograde flow along the abdominal and thoracic aorta. This potentially causes two specific complications: increase in left ventricle (LV) afterload^4^—which can cause LV dilation, secondary cardiac damage and pulmonary oedema—and differential hypoxaemia (DH).

DH occurs when the upper body, perfused with deoxygenated blood by the native heart and lungs, has a lower oxygen saturation than the lower body perfused with oxygenated blood from the ECMO circuit^5^. This happens in the case of concomitant pulmonary failure, which commonly ensues in the context of pulmonary oedema and/or pneumoniae. DH causes coronary and cerebral hypoxaemia and is a complication frequently encountered by ECMO clinicians. The effects of such DH on cerebral structures and integrity have been, however, poorly investigated.

We hypothesised that increasing V-A ECMO flow would lead to an optimized cerebral oxygen delivery and reduce brain injury risks assessed by biological, physiological and histological markers.

Thus, in a novel experimental model of severe cardiopulmonary failure supported by V-A ECMO, we aimed to study the impact of the ECMO flow on brain injury.

## Methods

Our methods are reported according to the ARRIVE guidelines^6^. Six Merino crossbred non-pregnant, female sheep, between the age of 1 and 3 years, weighing between 45 and 60 kg, were used in this study. Animals were sourced from the Commonwealth Scientific and Industrial Research Organisation (CSIRO) and housed at the Queensland University of Technology (QUT) Medical Engineering Research Facility, with ad libitum access to shade, food and water. The animals were fasted overnight prior to the experiment. The study was approved by the QUT Office of Research Ethics and Integrity (QUT: 1,800,000,337). All experiments were performed in accordance with NHMRC Australian Code of Practice for the Care and Use of Animals for Scientific Purposes and the Animal Care 8th Edition 2013 and Protection Act 2001 (QLD).

### Instrumentation, cardio-pulmonary failure and ECMO canulation

The experimental set-up used in this study has been published previously^7^ and details are available in Supplementary Materials. In summary, animals underwent general anaesthesia using propofol for induction and were placed on mechanical ventilation with the following settings: tidal volume (VT) of 8 mL kg^−1^, positive end-expiratory pressure (PEEP) of 5 cmH_2_O, respiratory rate (RR) and inspiratory fraction of oxygen (FiO_2_) adjusted to maintain an arterial partial pressure of oxygen (PaO_2_) between 60 and 100 mmHg, and an arterial partial pressure of carbon dioxide (PaCO_2_) between 35 and 45 mmHg. Anaesthesia was maintained using and midazolam and sufentanil. Cardiogenic shock was induced with multiple injections of 96% ethanol into the subepicardial layer of the left ventricle in order to obtain the following parameters (corresponding to cardiogenic shock definition according to the latest guidelines^1^): LV ejection fraction (EF) < 30%, systolic arterial pressure (SAP) < 90 mmHg for at least 10 min, arterial blood lactate > 4 mmol L^−1^ and/or urine output < 0.5 mL kg^−1^ h^−1^. Acute respiratory failure (ARF) was induced by reducing VT to 4 mL kg^−1^, PEEP to 0 cmH_2_O, RR to 5 per min and FiO_2_ to 21% to achieve a PaO_2_ below 60 mmHg and/or an arterial saturation of oxygen (SaO_2_) below 60%.

A detailed description of the ECMO equipment and set-up has been previously reported^7^ and is detailed in Supplementary Materials. Animals were cannulated using a 19-Fr right jugular venous access and a 15-Fr femoral arterial return cannula, the positions of which were assessed using fluoroscopy to obtain the tip of the access cannula in the inferior vena cava and the return cannula in the abdominal aorta below the renal arteries. Thereafter and until randomization, ECMO was started and set as follows: pump speed adjusted to achieve a flow rate of 1 L min^−1^ (i.e. the “ECMO flow” which corresponds to the blood flow at the end of the arterial tip), and fresh gas flow set at 2 L min^−1^ (i.e. the amount of gas necessary to adjust PaCO_2_). Blender was set to deliver 100% oxygen throughout the experiment.

### Randomized interventions

After instrumentation, sheep were randomly allocated to one of the following groups (n = 3 per group):

1. Low flow (LF): ECMO was set at 2.5 L min^−1^ to ensure that the brain was entirely perfused by the native heart and lungs, while V-A ECMO flow only reached the abdominal aorta close to the diaphragm^8^;
2. High flow (HF): ECMO was set up at 4.5 L min^−1^ to ensure that the brain was partially perfused by the ECMO.

**Figure 1:**
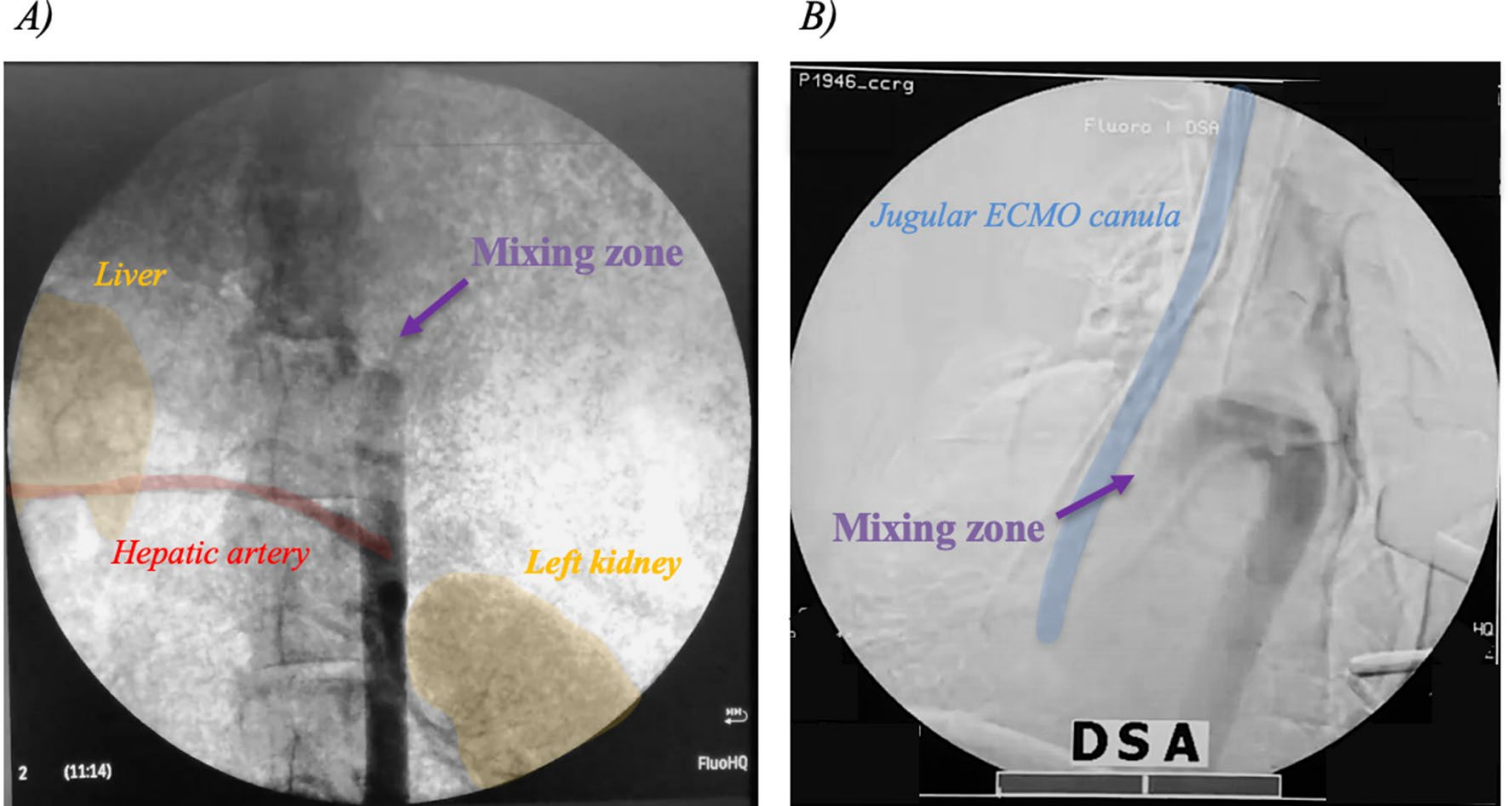
X-ray images of the mixing zone. Legend: The mixing zone corresponds to the zone where the LV and the ECMO meet. (**A**) Low flow: the mixing zone is in the abdominal aorta above the hepatic artery. (**B**) High flow: the mixing zone is in the ascending aorta (the whole aortic arch is opacified). ECMO = extracorporeal membrane oxygenation; LV = left ventricle.

The position of the mixing zone along the aorta, where LV and ECMO flows blend, was confirmed with fluoroscopy by infusing 40–60 mL of contrast medium (Ultravist® 370, iopromide, Bayer, USA) in the systemic circulation, via the lateral port of the arterial return cannula (Fig. 1, supplementary videos online). All other parameters were kept constant during the experiment.

**Figure 2:**
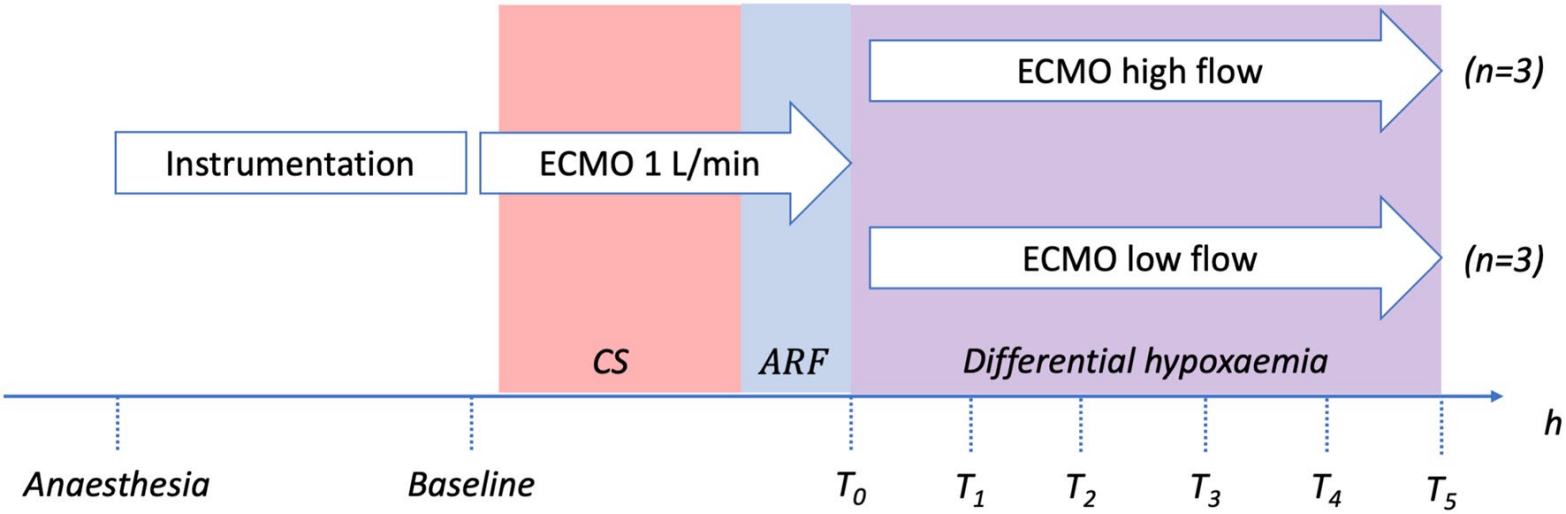
Study design and protocol. Legend: Instrumentation corresponds to anesthesia, establishing invasive monitoring and ECMO cannulation which took approximately 3 to 4 h. CS corresponds to the induction of cardiogenic shock by repeated injection of 96% ethanol in the LV. ARF corresponds to the induction of hypoxemia. Therefore, T_0_ is the time where differential hypoxemia (CS + ARF) criteria were met. T_1_ to T_5_ correspond to the study period, during which each group received either a low flow (2.5 L min^−1^) where brain is perfused entirely by the native heart and lungs, or a high flow (4.5 L min^−1^) where brain is partially perfused by the ECMO. ARF = acute respiratory failure, CS = cardiogenic shock, ECMO = extracorporeal membrane oxygenation, LV = left ventricle.

*Baseline* was defined as the first assessment after one hour from completion of sheep instrumentation, and *T*_0_ the time upon randomisation when differential hypoxaemia (i.e., CS + ARF) criteria were met. After randomisation, data was collected hourly until completion of the experiment after five hours. (Fig. 2).

### Cerebral assessments

We appraised effects of intervention on the brain using four distinct methods: haemodynamics, oxygenation, metabolism, and histology. For this purpose, during the instrumentation phase, after exposing the skull we drilled two burr holes on the right side of the skull, 5 mm lateral to the sagittal suture, from either side of the lambdoid suture.

- *Brain haemodynamics.* We continuously monitored cerebral perfusion pressure (CPP) (defined as mean arterial pressure (MAP) minus intracranial pressure (ICP)), and regional flow using laser-doppler velocity (OxyFlo, Oxford Optronics, Oxford, UK).
- *Brain oxygenation.* We measured non-invasive regional saturation (rSO_2_) obtained via near-infrared spectroscopy (NIRS) sensors placed on the frontal lobe (ForeSight Elite, Edwards Lifesciences, Nyon, Switzerland). Invasive oxygen saturation was monitored using oxygen tension (PbTO_2_) (OxyLite, Oxford Optronics). A change of 25% in the baseline values of rSO_2_ was considered abnormal, and these values were compared with rSO_2_ measured in the lower limb (opposite from the ECMO canulation), which served as reference.
- *Brain metabolism.* We performed microdialysis (CMA Microdialysis AB, Stockholm, Sweden) using a 20,000 Dalton cut-off perfused with CNS perfusion fluid (Microdialysis AB) at a rate of 0.3 μ L min^−1^. Micro-dialysates samples were collected hourly in capped microvials. Lactate, pyruvate, and glucose levels were monitored hourly using a CMA-600 microdialysis analyser (CMA Microdialysis AB). Based on the 2014 Consensus statement from International Microdialysis Forum^9^, we considered as abnormal the following values: glucose < 0.8 mmol L^−1^, lactate > 4 mmol L^−1^, pyruvate > 200 mmol L^−1^, lactate/pyruvate ratio (LPR) > 25. We kept systemic glucose and CO_2_ constant to ensure that variation in microdialysis parameters be related to cerebral flow and oxygenation. We considered the following metabolic patterns: normal (LPR ≤ 25 and lactate ≤ 4), ischemia (LPR > 25, lactate > 4, pyruvate ≤ 200), mitochondrial dysfunction (LPR > 25, lactate ≤ 4, pyruvate > 200).
- *Brain histology.* Animals were euthanized at the end of the experiment using 150 mg/kg of phenobarbitone (Lethabarb®), and their brains were immediately harvested to confirm appropriate position of intra-cerebral catheters and histological analysis. Brain tissue was fixed in 10% buffered formalin for at least 48 h, processed and embedded in paraffin. Sections (5 µm) were stained with haematoxylin and eosin and examined by light microscopy by a specialist veterinary pathologist (CP) blinded to the group allocation. Each sample consisted of eight sections (four from the frontal lobe and four from the parietal lobe) and ten fields per section were analysed. Seven parameters were then evaluated to score brain injury: neuronal shrinkage and hyperacidophilia, spongy state, congestion, perivascular oedema, perivascular haemorrhages, neutrophilic inflammation degree, and fibrin thrombi. A semi-quantitative analysis was performed with values being 0 (no lesion observed), 1 (< 5 lesions per field) or 2 (≥ 5 lesions per field) for each parameter (Supplementary Table 1).

### Kidney assessments

We inserted a microdialysis probe, invasive oxygen tension (PkO_2_) and flow probes (OxyLite/OxyFlo, Oxford Optronics) into the renal cortex through left mini-laparotomy. Kidney was monitored hourly and post-mortem histology was carried out. Nine parameters were then evaluated to score kidney injury: two in the glomeruli (fibrin and dilation of the Bowman’s space), four in the tubules (dilation, swelling, necrosis and hyaline casts presence within the lumen), and three in the interstitium (inflammation, congestion, and haemorrhage). A semi-quantitative analysis was performed with values being 0 (no lesion observed) to 2 (maximum lesions observed) for each parameter. (Supplementary Table 2).

### Outcomes

- *Primary outcome*
  Primary outcome was cerebral damage evaluated through the global cerebral injury score.
- *Secondary outcomes*

Secondary outcomes were:

- Brain metabolism parameters (glucose, pyruvate, lactate and LPR);
- Kidney histological assessment;
- Kidney metabolism parameters (glucose, pyruvate, lactate and LPR).

### Statistical analysis

For all tests, normality was tested with Kolmogorov–Smirnov test, and data was expressed as mean ± standard deviation. Data was expressed as median ± interquartile range when not distributed normally. Data was analysed using linear mixed effects model with a Geisser-Greenhouse correction, with time and group as fixed effects and sheep as random effect. Analysis was followed by multiple comparisons test with Tukey correction, both for differences between groups (high flow vs low flow) and along time (all time points vs T_0_). Histological analyses were considered as categorical variables and compared with a chi-squared test. All statistical analyses were two-sided, with a significance level set at 0.05, and were performed using GraphPad Prism 9 (GraphPad Software, San Diego, CA, USA).

## Results

All sheep survived procedures with no complications and completed follow-up. Systemic glucose, and carbon dioxide levels were kept constant throughout the experiment.

### Cardiopulmonary failure characteristics

All sheep achieved pre-defined CS criteria with no statistical differences between groups or timepoints (Table 1). Hypoxaemia was successfully achieved in all sheep with a mean PaO_2_ at randomization of 48 ± 15 versus 42 ± 11 mmHg in the low flow and high flow group, respectively (*p* > 0.99).

**Table 1:** Hemodynamic variables at different timepoints for each study group.

	Low flow (N = 3)	High flow (N = 3)
Heart rate (beats min^*−*1^)		
Baseline	91 ± 7	110 ± 18
T0	86 ± 17	85 ± 56
T1	91 ± 21	68 ± 23
T2	96 ± 22	80 ± 11
T3	89 ± 17	78 ± 10
T4	89 ± 1	96 ± 27
T5	92 ± 8	92 ± 6
SAP (mmHg)		
Baseline	65 ± 13	82 ± 7
T0	69 ± 8	81 ± 39
T1	69 ± 4	86 ± 20
T2	72 ± 7	90 ± 13
T3	70 ± 7	89 ± 11
T4	73 ± 4	93 ± 15
T5	77 ± 6	94 ± 17
MAP (mmHg)		
Baseline	58 ± 1	69 ± 7
T0	62 ± 3	70 ± 45
T1	66 ± 6	81 ± 14
T2	66 ± 6	85 ± 11
T3	63 ± 2	81 ± 11
T4	70 ± 8	81 ± 6
T5	72 ± 2	84 ± 11
Pulse pressure (mmHg)		
Baseline	17 ± 6	19 ± 1
T0	14 ± 9	16 ± 9
T1	7 ± 4	8 ± 8
T2	12 ± 7	8 ± 6
T3	11 ± 8	11 ± 1
T4	12 ± 6	18 ± 17
T5	11 ± 10	15 ± 12
Lactate (mmol L^*−*1^)		
Baseline	1.5 ± 0.7	1.6 ± 0.4
T0	6.0 ± 0.8	5.7 ± 1.6
T1	7.3 ± 1.0	8.0 ± 3.5
T2	7.3 ± 1.1	9.0 ± 4.8
T3	7.3 + 1.8	9.1 ± 5.9
T4	7.7 ± 3.3	8.6 ± 7.3
T5	8.0 ± 4.3	8.4 ± 8.3
LVEF (%)		
Baseline	46 ± 18	47 ± 5
T0	28 ± 7	16 ± 6

*LVEF* left ventricular ejection fraction; *MAP* mean arterial pressure; *SAP* systolic arterial pressure.

### Low flow versus high flow characteristics

**Figure 3:**
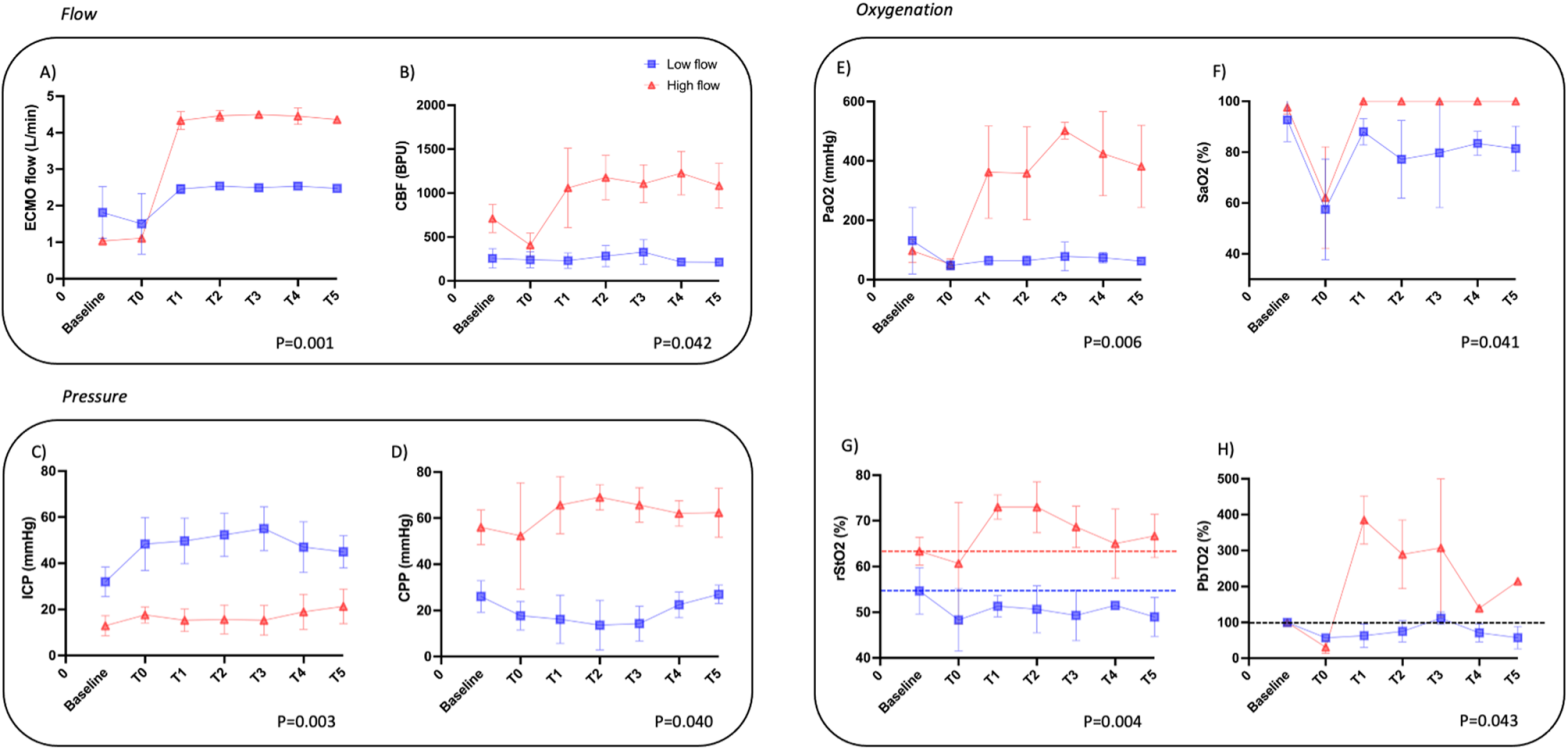
Flow, pressure, and oxygenation parameters at different timepoints for each study group. Legend: (**A**) ECMO flow in L min^−1^; (**B**) local cerebral blood flow in blood per units (BPU) obtained by OXYFLOW; (**C**) ICP in mmHg; (**D**) CPP in mmHg; (**E**) PaO_2_ in mmHg; (**F**) SaO_2_ in %; (**G**) rStO_2_ in % obtained by NIRS; (**H**) PbTO_2_ variation from baseline (in %) obtained by OXYLITE. Arterial blood gases were drawn from the right radial artery. Red lines represent results from the high flow group and blue lines from the low flow group. Dotted lines for rStO_2_ and PbTO_2_ represent the initial (“baseline”) levels. Data are represented as mean ± SD. BPU = blood per units; CPP = cerebral perfusion pressure; ECMO = extracorporeal membrane oxygenation; ICP = intracranial pressure; NIRS = near-infrared spectroscopy; PaO_2_ = partial arterial pressure of oxygen; PbTO_2_ = partial brain oxygen tension; SaO_2_ = arterial saturation of oxygen; rStO_2_ = tissue oxygen saturation.

Figure 3 shows data obtained in terms of flow (V-A ECMO flow and cerebral brain flow), pressure (ICP and CPP) and oxygenation (PaO_2_, SaO_2_, rSO_2_ and PbTO_2_) in both groups during experimentation. V-A ECMO flows achieved predefined values (mean flow of 2.50 ± 0.06 and 4.42 ± 0.15 L min^−1^, respectively) which translated into significantly different cerebral blood flows of 205 ± 76 and 970 ± 400 BPU respectively (*p* = 0.042). Cerebral oxygenation parameters were also significantly higher in the high flow when compared to low flow group in terms of PaO_2_ (*p* = 0.006), SaO_2_ (*p* = 0.041), rSO_2_ (*p* = 0.004) and PbTO_2_ (*p* = 0.043). At baseline, animals had similar PbTO_2_ values in both the low (16.72 ± 19.7 mmHg) and the high flow (11.94 ± 5.7 mmHg) group (*p* = 0.96). SaO_2_ was 82.6 ± 12.6% in the low flow group throughout the experiment.

### Primary outcome

**Figure 4:**
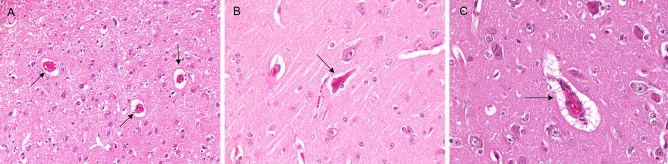
Hematoxylin/eosin—stained sections of the brain. Legend: Examples of scores brain pathologies: (**A**) vascular congestion (arrow); (**B**) neuronal shrinkage (arrow); (**C**) congested blood vessel with perivascular edema (arrow).

**Figure 5:**
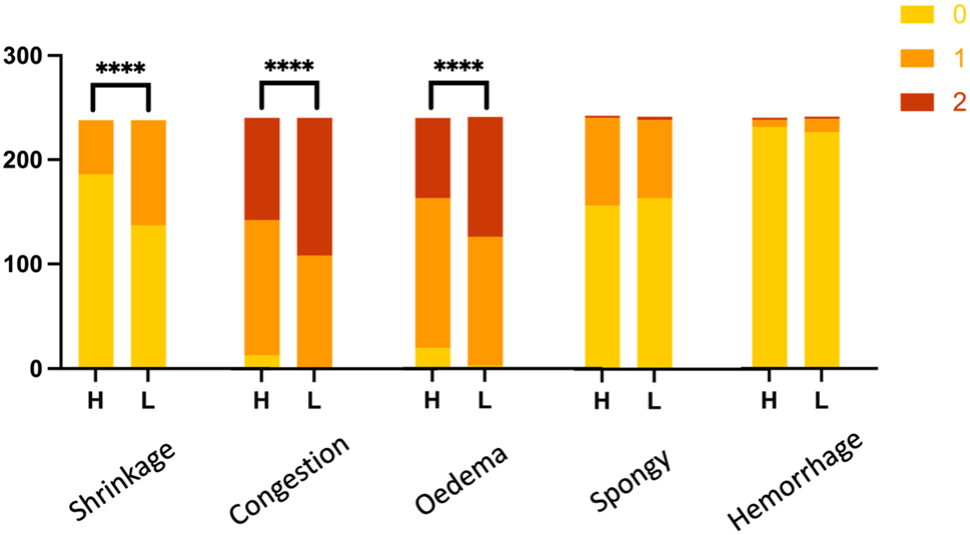
Histological scoring differences of the brain between low flow (L) and high flow (H) groups. Legend: Histograms represent the proportion of each score for all 240 samples (y-axis) analyzed. A minimum score of 0 (in yellow) corresponds to the absence of damage, whereas a maximum score of 2 (in red) corresponds to a high level of damage (> 5 lesions per field). Inflammation and thrombosis were not observed and are therefore not represented in this figure. **** *p* < 0.0001.

Cerebral global histological injury score was significantly higher in the low flow group compared to the high flow group (*p* = 0.0003). Histology showed some degrees of brain damage in both groups (Fig. 4.), except for inflammation and thrombosis which were absent. Neuronal shrinkage, congestion, and perivascular oedema were significantly higher in the low flow group (*p* < 0.0001) (Fig. 5).

### Secondary outcomes

**Figure 6:**
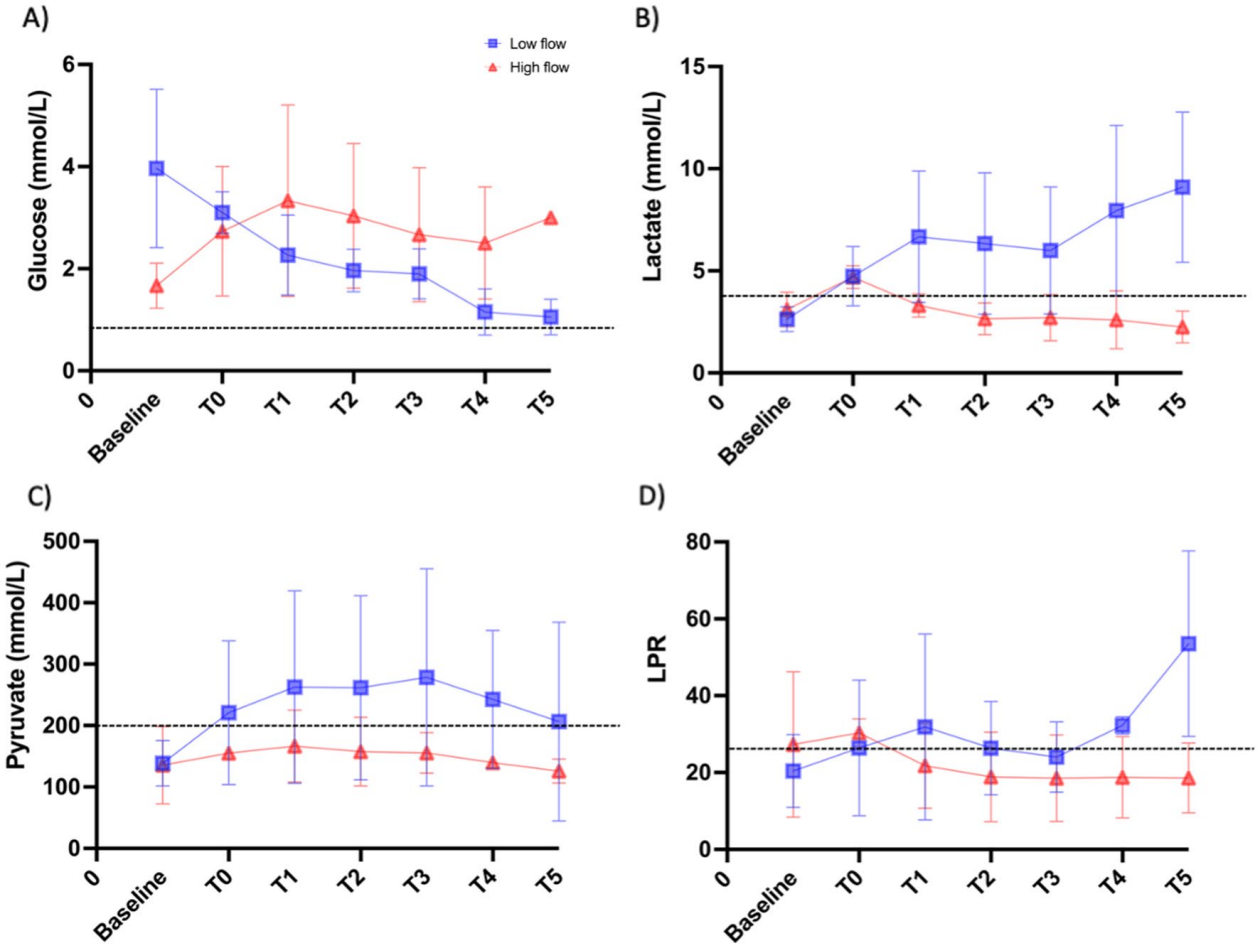
cerebral microdialysis values at different timepoints for each study group. Legend: Concentrations of (**A**) glucose (mmol L^−1^), (**B**) lactate (mmol L^−1^), and (**C**) pyruvate (mmol L^−1^), and (**D**) lactate-pyruvate ratio (LPR). Red lines represent results from the “high flow” group and blue lines from the “low flow” group. Dotted lines represent pathological thresholds as defined by the 2014 Consensus statement from International Microdialysis Forum^8^.

Brain metabolism parameters obtained from microdialysis are summarized in Fig. 6. Analysis found no significant difference between the two groups but values in the low flow group all reached predefined pathological threshold.

Kidney histopathological examination reported some degree of injury in both groups, especially vascular congestion (74%) and dilation of the Bowman’s space (50%). Cloudy swelling of the tubular epithelium was significantly higher in the high flow group (*p* < 0.0001) and accumulation of granular material/hyaline casts within the tubular lumen were significantly higher in the low flow group (*p* < 0.001). Kidney microdialysis showed no statistical difference between groups for glucose (*p* = 0.61) nor LPR (*p* = 0.95). (Supplementary Fig. 1 and Fig. 2).

## Discussion

Differential hypoxemia leads to significant complications in patients supported by VA-ECMO, with a decrease in cerebral oxygenation and therefore, damage to cerebral cells being one of the major challenges. Using a previously published cardio-pulmonary failure supported by VA-ECMO model, two clinically relevant ECMO flows were tested which translated into different degrees of differential hypoxaemia as shown by both invasive (PbTO_2_) and non-invasive (NIRS) cerebral oxygenation. Although limited by its sample size, this study demonstrated significantly less pronounced histological brain damage in the group receiving high flow support compared to the low flow group. This could be explained by relative conservation of the cerebral aerobic state as shown by the lower lactate levels and LPR in the cerebral microdialysis analysis.

Cerebral complications occurring while on V-A ECMO are classically described as acute vascular accidents (ischemic and/or haemorrhagic) or microbleeds^10^, reported between 13 and 20% in recent literature^11,12^. This is mostly due to the relative short time spent by patients on ECMO (few days to weeks). Nevertheless, a more chronic malperfusion and/or hypoxaemia could also participate in the cognitive decline seen in some ECMO survivors^13^. These complications are probably underestimated because of the lack of standardized reporting criteria and the broad variety of severity ranging from seizures to brain death^14^. In addition, it is not always possible to determine if those injuries were favoured by factors present before ECMO implantation, such as hypoxia. Nevertheless, these neurological complications are acknowledged to be responsible for a high morbidity and mortality.

Differential hypoxaemia corresponds to relative hypoxaemia in the upper body (perfused by the LV) compared to the lower body (perfused by the ECMO). Data regarding DH are lacking, which can be explained by several factors: (1) DH is a highly variable and dynamic condition, (2) multiple factors can contribute to these variations, such as the proportion between native LV and ECMO flows^15,16^, arterial cannula tip position^5^, and venous drainage position^17^, and (3) the carotids of most large animals (bovine, pigs, primates) depart from a common bi-carotid trunk (also known as *bovine arch*) making cerebral studies during VA ECMO less translatable to humans. As a result, it is unclear if DH creates additional complications by causing cerebral hypoxia^18^ or if a relative level of hypoxia is acceptable, as suggested by some authors^19^.

We investigated cerebral lesions using histology as our primary outcome. Of other possibilities to screen for cerebral damage, this choice was made for several reasons: magnetic resonance imaging is contra-indicated with ECMO, computerized tomography scan shows anomalies with a few hours delay, electroencephalography was not available in sheep. Thus, histology seemed the best choice to investigate early brain lesions. We used a multimodal cerebral monitoring comprising of oxygenation, pressure, and metabolism to explore potential mechanisms explaining these lesions.

Indeed, studying cerebral metabolism was essential as one could have argued that the increase in ECMO flow—shifting from a “low pulsatile hypoxemic flow” to a “high pulseless hyperoxemic flow”—could have induced additional damage similar to an ischemia–reperfusion-like syndrome with hyperoxia, as observed in resuscitated cardiac arrest, post-carotid stenosis revascularisation, traumatic brain injury or stroke^20,21^. In the latter situations, the negative effects of hyperoxaemia are thought to be generated via reactive oxygen species exacerbating the initial reperfusion injury via the recruitment of neutrophils, causing a disproportionate inflammatory response^22^. Effects of hyperoxaemia alone (without an initial brain injury) are currently debated as results are not equivocal^23–25^. A recent study found that hyperoxemia was independently associated with an increase in mortality in patients undergoing V-A ECMO for cardiogenic shock^26^.

We observed cerebral lesions after only a few hours spent in DH and a reduction of those when ECMO flow was increased. These results could be due to an increase in cerebral flow, oxygenation, or a combination of both. All our results are compatible with typical hypoxic-ischemic lesions: histological findings^27,28^, regional variation of NIRS, and microdialysis results^9,29^.

One last parameter was modified between low and high flow: pulsatility. Indeed, an increase in flow will increase LV afterload and mechanically decrease pulsatility, although we did not observe a significant change in pulse pressure (common definition of pulsatility). As no study has yet reported an impact of pulsatility *alone* on brain or circulating cells damage except for von Willebrand factor^30,31^, and given the fact that the arterial tip of the ECMO is almost facing the renal arteries, pulsatility *alone* is unlikely to explain changes in brain or kidney histology we observed.

We decided to use kidney as control for our experiment as they experienced ischemia–reperfusion injuries like the brain (from the induction to cardiogenic shock to the ECMO support) but without any hypoxaemia. Histology showed some degree of injury which are non-specific and could be related to several factors known to induced acute kidney injury such as ischemia–reperfusion, systemic inflammation or non-pulsatile flow^32,33^. Microdialysis found no difference between the two groups and in particular, we did not observe an increase in kidney damage with a higher flow. Overall, these results tend to confirm that brain lesions were triggered by differential hypoxaemia and not solely by ischemia–reperfusion.

This is the first experiment assessing the effect of the V-A ECMO flow consequences on the brain in a context of DH. We used a cardiogenic shock model developed specifically for this study^7^, allowing us to keep native LV output constant—where other CS models (especially acute myocardial infarction models) suffer from a decrease in LV cardiac output over time^34^. We confirmed arterial cannula tip position and mixing zone location for each animal using fluoroscopy which allowed our results to be consistent and robust. Our decision to use an ovine model was made to avoid bovine arch thus enhancing clinical translation of our results. Animals were randomised to avoid selection bias. We demonstrated that some degree of cerebral damage appeared already after 5 h of DH for upper body PaO_2_ levels that can be considered not “profound”, and that an increase in ECMO flow—a very common strategy used in case of DH—was effective in reducing these damages. Interestingly, even though systemic arterial lactate was relatively constant in both groups, brain lactate kept diminishing in the high flow group, suggesting a cerebral adaptation to DH in the high flow group. Nevertheless, DH complications could not be entirely reversed.

Apart from its small sample size, our study suffers several limitations. First, the follow-up period was relatively short (5 h). Nevertheless, we considered this to be clinically relevant as most patients would benefit from an intervention to reverse DH in such delays. Also, we were able to obtain significant results despite a short period of time, and temporal trends are in favour of the effect of treatment. Second, the effects of the position of the cannulas (i.e. superior vena cava or inferior vena cava for the drainage and a higher position for the return canula) were not investigated. Indeed, Hou et al.^17^ demonstrated that the position of the venous canula improved upper body oxygenation, yet this was not an objective in our study and venous cannulas were systematically in the inferior vena cava. Third, to better characterise brain damage, neurobiomarkers such as neuron specific enolase (NSE), glial fibrillary acidic protein (GFAP) or S-100B would have been beneficial. However, antibodies assays are not all available in sheep, have not been fully investigated, and their use/threshold have been poorly investigated in patients undergoing ECMO^35–37^. Fourth, we have not been able to study cerebral autoregulation, an important parameter to fully understand brain hemodynamic, as transcranial doppler is not feasible in sheep. Finally, our model investigated previously healthy, young, female animals which may be a limitation to generalisation of results.

Our conclusion—“protect the brain”—may be simple, but advocates for continuous neuromonitoring during V-A ECMO, which is not currently performed in clinical practices^38^. In the absence of better tools, we suggest using frontal NIRS to help clinicians guide their behaviour in case of suspected differential hypoxaemia. In case of differential hypoxaemia, increasing ECMO flow could be considered an option and hyperoxia should be avoided. Nevertheless, increasing the ECMO flow comes with a price of increasing LV afterload and should only be considered as a safety procedure while waiting for a sustainable intervention. Since we did not assess mortality or long-term outcome, it is not guaranteed that the benefits of improving cerebral oxygenation overcome the LV damage caused by afterload increase. Indeed, a recent meta-analysis found that LV unloading significantly decreased mortality^39^.

## Conclusion

Among animals undergoing peripheral V-A ECMO support, differential hypoxaemia lead to cerebral damage after only a few hours, which could be partially corrected by increasing ECMO flow. Neuromonitoring of these patients is therefore of great importance. This strategy could help clinicians experiencing differential hypoxaemia although left ventricle afterload should be carefully monitored.

## Supplementary Information

Supplementary Information 1.

Supplementary Video 1.

Supplementary Video 2.

## Data Availability

The datasets used and/or analysed during the current study available from the corresponding author on reasonable request.

## Abbreviations

ARF: Acute respiratory failure
CPP: Cerebral perfusion pressure
CS: Cardiogenic shock
DH: Differential hypoxaemia
ECMO: Extracorporeal membrane oxygenation
EF: Ejection fraction
FiO_2_: Inspired fraction of oxygen
HF: High flow
ICP: Intracranial pressure
LF: Low flow
LPR: Lactate-pyruvate ratio
LV: Left ventricle
MAP: Mean arterial pressure
NIRS: Near-infrared spectroscopy
PaCO_2_: Arterial partial pressure of carbon dioxide
PaO_2_: Arterial partial pressure of oxygen
PbTO_2_: Partial brain oxygen tension
PEEP: Positive end-expiratory pressure
RR: Respiratory rate
rStO_2_: Regional saturation of oxygen
SAP: Systolic arterial pressure
V-A: Veno-arterial
VT: Tidal volume
