# Supplementary material for: Effect of flow change on brain injury during an experimental model of differential hypoxaemia in cardiogenic shock supported by extracorporeal membrane oxygenation
Source: Sci Rep. 2023 Mar 10;13:4002. doi: 10.1038/s41598-023-30226-6 (PMC10006234; doi:10.1038/s41598-023-30226-6)
Supplement: Supplementary file 1 — Supplementary Information 1. [file 41598_2023_30226_MOESM1_ESM.docx]

Supplementary data

**Effect of flow change on brain injury during an experimental model of differential hypoxaemia in cardiogenic shock supported by extracorporeal membrane oxygenation**

**Detailed cardiogenic shock protocol**

**Detailed extracorporeal membrane oxygenation (ECMO) protocol**

**Supplementary Table 1:** scoring system for the histological assessment of the brain

**Supplementary Table 2:** scoring system for the histological assessment of the kidney

**Supplementary Figure 1:** kidney microdialysis results

**Supplementary Figure 2:** kidney histology results

**Supplementary Video 1 “MZ down”:** example of mixing zone location in the low flow group

**Supplementary Video 2 “MZ up”:** example of mixing zone location in the high flow group

**Detailed cardiogenic shock protocol**

Amiodarone (6 mg.kg*^-1^*) and lidocaine (8 mg.kg*^-1^*) were administered prophylactically to avoid ventricular arrhythmias during the procedure. A left lateral mini-thoracotomy was performed at the 5th–6th intercostal space to expose the heart. Multiple injections of 96% ethanol were carried out into the subepicardial layer of the anterior, inferior and lateral left ventricle (LV) free wall, parallel and superficial to its surface.

Global and regional contractility was sequentially assessed after 30 injections, and every 10 injections thereafter, through direct epicardial echocardiography.

In addition, after visual echocardiography assessment, we evaluated the presence of cardiogenic criteria, as listed below, were met to cease ethanol injections:

- Left ventricle ejection fraction below 30%;
- Reduction of arterial systolic blood pressure below 90 mmHg for more than 10 min;
- Arterial blood lactate > 4 mmol.L*^-1^*;
- Urinary output < 0.5 mL/kg/h.

**Detailed extracorporeal membrane oxygenation (ECMO) support**

The ECMO circuit comprised a Rotaflow console, centrifugal pump, tubing and a low resistance oxygenator (Quadrox D, Maquet Cardiopulmonary GmbH, Germany) primed with 0.9% saline and heated at 37 °C via a heater-cooler machine (Getinge AB, Sweden).

Animal was placed in the supine position for cannulation. The left femoral artery was exposed surgically through a cut-down technique. After achievement of activated clotting time ≥ 200 s with a bolus of unfractionated heparin, a guidewire was inserted into the right external jugular vein sheath and a venous multi- stage 19-Fr cannula (Maquet Cardiopulmonary GmbH, Germany) was advanced down to the inferior vena cava under fluoroscopic guidance. Finally, a 15-Fr arterial cannula (Maquet Cardiopulmonary GmbH, Germany) was inserted through the exposed left femoral artery and advanced up to the descending aorta.

After cannulation, ECMO pump speed was increased to 1500 RPM prior to clamp release and was set as follows: pump speed adjusted to achieve a flow rate of 1 L.min*^-1^*, fresh gas flow set at 2 L.min*^-1^* and blender set to deliver 100% oxygen at all times.

**Supplementary Table 1:** scoring system for histological assessment of the brain

| Observations | Score 0 | Score 1 | Score 2 |
| --- | --- | --- | --- |
| Neuronal shrinkage, hyperacidophilia  (20x) | none | < 10 cells affected | ≥ 10 cells |
| Spongy state  (20x) | none | < 50% tissue | ≥ 50% tissue |
| Congestion  (20x) | none | < 50% field | ≥ 50% of the field |
| Perivascular oedema  (10x) | none | < 50% blood vessels | ≥ 50% blood vessels |
| Perivascular haemorrhages  (10x) | none | < 50% blood vessels | ≥ 50% blood vessels |
| Neutrophilic inflammation  (20x) | none | < 20 neutrophils | ≥ 20 neutrophils |
| Fibrin thrombi  (10x) | none | 1 thrombus | > 1 thrombi |

**Supplementary Table 2:** scoring system for histological assessment of the kidney

| Observations | Score 0 | Score 1 | Score 2 |
| --- | --- | --- | --- |
| **Glomeruli** | | | |
| Fibrin thrombi within the capillary loops (40x) | none | 1 thrombus | ≥ 2 thrombi |
| Dilation of the Bowman’s space with accumulation of granular material (10x) | none | < 50% glomeruli | ≥ 50% glomeruli |
| **Proximal and distal tubules (cortex)** | | | |
| Cloudy swelling  (20x) | none | < 50% epithelial cells | ≥ 50% epithelial cells |
| Necrosis  (20x) | none | < 50% epithelial cells | ≥ 50% epithelial cells |
| Granular material or hyaline casts within the lumen (20x) | none | < 50% tubules | ≥ 50% tubules |
| **Interstitium** | | | |
| Acute inflammation  (20x) | none | < 50% inflammatory cells | ≥ 50% inflammatory cells |
| Congestion  (20x) | none | < 50% tissue affected | ≥ 50% tissue affected |
| Haemorrhage  (20x) | none | < 50% tissue affected | ≥ 50% tissue affected |

**Supplementary Figure 1:** kidney microdialysis


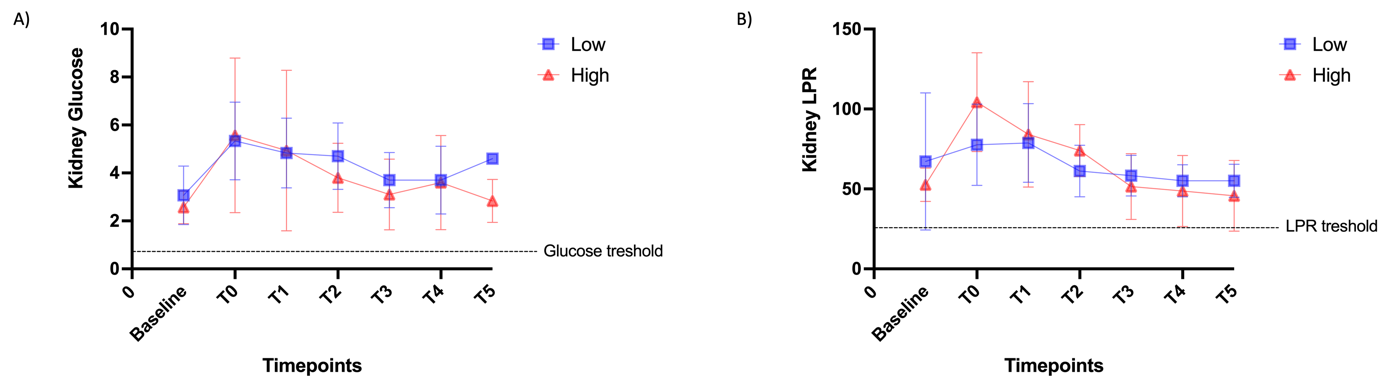


**Legend:** *kidney microdialysis* *values at different timepoints for each study groups. Concentrations of A) glucose (mmol.L^-1^) and B) lactate-pyruvate ratio (LPR). Dotted lines represent pathological thresholds as defined by the 2014 Consensus statement from International Microdialysis Forum*^7^*. Red lines represent results from the high flow group and blue lines from the low flow group.*

**Supplementary Figure 2:** histological assessment of the kidney


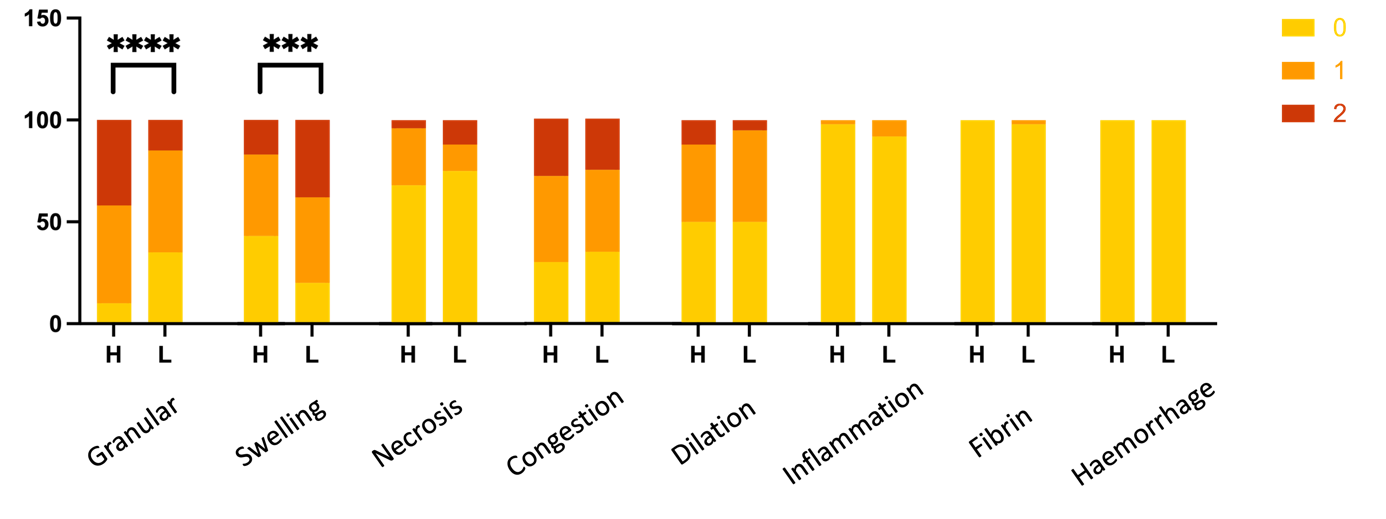


**Legend:** *kidney histological scoring differences between low flow (L) and high flow (H) groups. Histograms represent the proportion of each score for all samples analyzed. A minimum score of 0 (in yellow) corresponds to the absence of damage, whereas a maximum score of 2 (in red) corresponds to a high level of damage. **** P<0.0001, *** P<0.001.*

**Supplementary Video 1 “MZ down”:** example of mixing zone location in the low flow group

*Legend:* video of the contrast agent injected via the lateral port of the ECMO canula showing, in fluoroscopy, the mixing zone location in the abdominal aorta

**Supplementary Video 2 “MZ up”:** example of mixing zone location in the high flow group

*Legend:* video of the contrast agent injected via the lateral port of the ECMO canula showing, in fluoroscopy, the mixing zone location in the ascending aorta
